# Prevalence and Factors Affecting Appropriate Inhaler Use in Elderly Patients with Chronic Obstructive Pulmonary Disease: A Prospective Study

**DOI:** 10.3390/jcm12134420

**Published:** 2023-06-30

**Authors:** Le Khac Bao, Nguyen Dang Khoa, Le Thi Kim Chi, Nguyen Tuan Anh

**Affiliations:** 1 Department of Internal Medicine, University of Medicine and Pharmacy at Ho Chi Minh City, 217 Hong Bang, Ward 11, District 5, Ho Chi Minh City 700000, Vietnam; lekhacbao@ump.edu.vn (L.K.B.); ndkhoa.nt.noi.19@ump.edu.vn (N.D.K.); kimchidhyd@ump.edu.vn (L.T.K.C.); 2 Department of Respiratory, University Medical Center Ho Chi Minh City, 215 Hong Bang, Ward 11, District 5, Ho Chi Minh City 700000, Vietnam

**Keywords:** appropriate inhaler user, inhaler, elderly, COPD

## Abstract

Background: Chronic obstructive pulmonary disease (COPD) mainly affects individuals aged 60 and older. The proper use of inhalers is crucial for managing COPD. This study aimed to evaluate the prevalence and factors affecting the appropriate use of inhalers among elderly patients with COPD. Methods: We enrolled 91 elderly patients with COPD admitted to the Department of Respiratory, University Medical Center HCMC between October 2020 and May 2021. Patients who were capable of using the inhaler would have their inhaler usage recorded through video footage. Two respiratory experts carefully analyzed 133 video-recorded demonstrations for evaluation purposes. Results: 18.7% of the patients demonstrated the correct inhaler technique. Pressurized metered dose inhaler (pMDI) and Turbuhaler had the lowest documented correct usage rates (11.9% and 10.0%, respectively). Two critical steps, namely “holding breath for about five seconds or as long as comfortable” and “breathing out gently,” were commonly performed incorrectly when using pMDI, Respimat, Breezhaler, or Turbuhaler. Multivariable logistic regression analysis showed that lower mMRC scores (AOR = 5.3, CI 1.1–25.5, *p* = 0.037) and receiving inhaler instruction within the past three months (AOR = 5.2, CI 1.3–20.1, *p* = 0.017) were associated with increased odds of using the inhaler correctly. Conclusions: Our study found that less than 20% of elderly patients with COPD use inhalers correctly. Common errors include inadequate breath-holding and gentle exhalation. mMRC scores and recent inhaler instruction were predictors of proper use. These findings can aid clinicians in improving inhaler management for elderly patients with COPD.

## 1. Introduction

Chronic obstructive pulmonary disease (COPD) is a heterogeneous respiratory disease that manifests various clinical presentations, including chronic dyspnea, cough, sputum production, and recurrent exacerbations [1,2]. It is typically associated with airway abnormalities (e.g., bronchiolitis) and alveolar destruction, leading to persistent and progressive worsening airflow limitation [1]. Age is a significant risk factor for COPD, and the age-associated impairment of pulmonary function contributes to deteriorating disease symptoms [1,3]. People over 60 years old have the highest incidence of COPD [1,4], approximately two to three times higher than patients under 60 [3].

Inhalation therapy remains essential for COPD [1]. It allows medications to arrive directly at the affected bronches, thereby minimizing potential systemic side effects [5]. However, several studies have indicated that managing COPD with inhaled medications has yet to succeed due to suboptimal medication use, with non-adherence to treatment and incorrect use of inhalers being the principal factors [6,7,8]. Despite the efforts of healthcare workers and patients, the rate of correct inhaler uses ranges from 17% to 79% and has remained unchanged over the past 40 years [5,9,10,11,12].

Multiple factors have been associated with the correct use of inhalers, including educational level, income, and comorbidities such as heart failure, coronary artery disease, lung cancer, and depression [13,14,15,16,17]. Furthermore, patients with more severe COPD, higher levels of airflow limitation as measured by spirometry, and more frequent exacerbations in the past 12 months are at significant risk of misusing inhalers [7,11,18]. However, these studies have been conducted on different groups of patients, with subjective judgment by only one expert viewer. In addition, the rapid development of new inhaler devices such as Breezhaler and Accuhaler provides diverse options for various patient groups. In exchange for the benefits, many new devices affect the knowledge and skills of healthcare professionals and patients who have become accustomed to older devices [19]. The elderly population is vulnerable and is more likely to acquire cognitive or physical disorders affecting their ability to learn and maintain necessary skills [20,21,22]. Although previous research uncovered the causes behind increased inhaler misuse in elderly patients, there has not been a comprehensive analysis of the proportion and types of mistakes made with different inhalers [8,23,24]. Therefore, the study aimed at identifying the accuracy of inhaler usage among patients with COPD of more than 60 under multiple expert reviews. Additionally, the study revealed the most critical inhaler usage steps prone to mistakes and factors that increase the likelihood of correct inhaler usage in elder adults with COPD.

## 2. Materials and Methods

A prospective study was conducted at the Department of Respiratory, University Medical Center Ho Chi Minh City, Vietnam, from September 2020 to May 2021.

### 2.1. Inclusion Criteria

Our study enrolled patients over 60 years old diagnosed with COPD and admitted to the Department of Respiratory, University Medical Center Ho Chi Minh City. The diagnosis of COPD was established according to the Global Initiative for Chronic Obstructive Lung Disease (GOLD) guidelines [1], including (i) chronic cough and progressive dyspnea during exertion; (ii) history of smoking or exposure to harmful dust; and (iii) previous respiratory function tests that showed non-fully reversibility of airflow limitation (fixed ratio of forced expiratory volume in one second [FEV_1_]/forced vital capacity [FVC] < 0.7 post-bronchodilation test). Each patient had been using one or more types of inhalers at their residence for at least one month, either continuously or as needed, to treat COPD.

### 2.2. Exclusion Criteria

Patients with the following characteristics were excluded: (i) insufficient mental capacity to provide a history and report their past medical conditions; (ii) those who cannot maintain a SpO_2_ level of over 92% when breathing room air for more than 12 h; (iii) those currently experiencing acute myocardial infarction, acute stroke, or delirium; and (iv) patients who declined to participate in the study.

### 2.3. Sample Size

Thus, the sample size was calculated based on the proportion of correct use of all inhaler devices by all study participants without separately calculated for each specific type of inhaler. Chau et al. conducted a study in Vietnam on a COPD population similar to ours, which included hospitalized patients, and reported a correct inhaler use rate of 24.3% [11]. We used the following formula to calculate the sample size based on a proportion:

$$n=\frac{{Z^{2}}_{1-\alpha /2}\times p(1-p)}{d^{2}}$$

- *Z*_1−*α*/2_ is the corresponding coefficient for a 95% confidence interval (CI), where α = 5%.
- *p* is the proportion of correct inhaler use; *p* = 24.3% based on Chau’s study [11].
- *d* is the standard error. We chose *d* = 9%.

Therefore, our study would require a minimum of 88 participants.

### 2.4. Study Design

**Figure 1:**
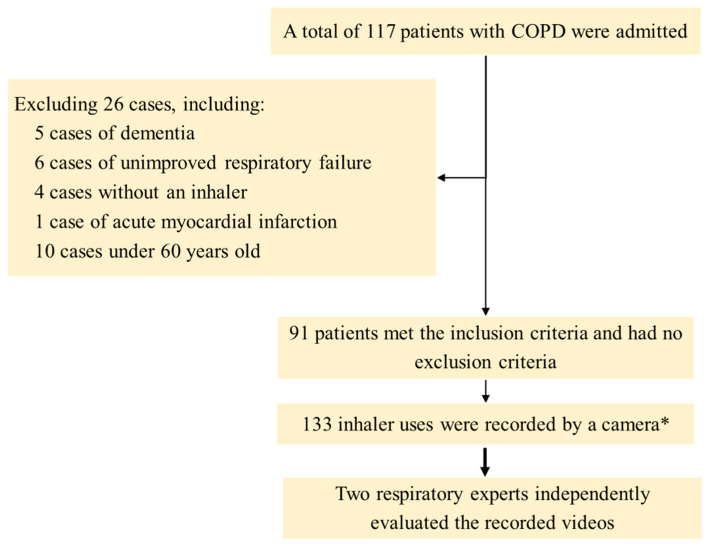
Study protocol. * The number of inhalers used may exceed the number of patients, as a single patient may use multiple types of inhalers simultaneously at home. COPD: chronic obstructive pulmonary disease.

We collected data on 117 patients with COPD admitted to the hospital, of whom 26 cases were excluded, including 5 cases of dementia, 6 cases of unimproved respiratory failure, 4 cases without an inhaler, 1 case of acute myocardial infarction, and 10 cases under 60 years old. Therefore, a total of 91 cases were included in the analysis. After receiving treatment for exacerbation of COPD, patients who are able to maintain a SpO_2_ level of over 92% when breathing room air for more than 12 h will be required to perform the technique of using the available inhalers at home. If the patient had multiple inhalers of the same type, such as a pressurized metered dose inhaler (pMDI) containing fluticasone propionate/salmeterol and a pMDI containing salbutamol, only one representative device was used. If the patient used multiple types of inhalers, such as a soft mist inhaler (SMI) containing tiotropium and a dry powder inhaler (DPI) containing fluticasone propionate/salmeterol, they would be required to perform their technique for using both devices in separate instances. During the procedure, patients were recorded using digital devices with a resolution of at least 720 pixels in a well-lit environment, ensuring a clear view of the patient’s nose, mouth, and abdomen. The videos were blurred from the eye area upwards and sent to two respiratory experts to separately evaluate the accuracy of inhaler use using checklists. The study protocol is illustrated in Figure 1.

### 2.5. Definition of Variables

The education level was defined as follows: low-level: pre-primary, primary, and lower secondary; middle-level: upper secondary and post-secondary; high-level: first-stage tertiary and second-stage tertiary [25]. Comorbidities were diagnosed during patients’ hospitalization using international guidelines, including asthma [26], bronchiectasis [27], chronic coronary syndromes [28], and chronic heart failure [29]. The modified medical research council (mMRC), COPD assessment test (CAT) scales, GOLD grades of airflow limitation, and COPD group definition are used following GOLD guidelines [1]. “Facial deformity” is defined to include cases where patients with facial deformities resulting from trauma, facial surgery, or congenital underdeveloped jaw, may have difficulties in achieving a proper seal, leading to medication leakage during inhalation, even though they understand and can perform all other steps correctly. We used inhaler technique checklists for pMDI, pMDI spacer plus, Accuhaler, Breezhaler, Respimat, and Turbuhaler based on the guidance of the National Asthma Council of Australia. The guidance can be found online at the following website: https://www.nationalasthma.org.au/living-with-asthma/resources/health-professionals/charts/inhaler-technique-checklists (accessed on 20 May 2023). The Respimat spacer plus was used in the study conducted by Takashi et al. [30]. However, no inhaler technique checklists were available for the Respimat spacer plus. Therefore, we made a checklist for using Respimat spacer plus (mainly based on the checklist for using pMDI spacer plus and Respimat), which can be found in Supplementary Data.

The definition of the critical steps in using an inhaler lacks consistency in the literature. However, a systematic review conducted by Usmani et al. defines critical steps as actions that impact the lung deposition of the inhaled drug, leading to minimal or no drug reaching the lungs [31]. We define a step as critical when more than half of the studies in the systematic review agree on it [31]. If a patient performs any critical step incorrectly in the inhaler technique checklist, it will be defined as an incorrect user [18]. Critical steps were defined based on inhaler technique checklists under the guidance of the National Asthma Council of Australia, which can be found in Supplementary Data.

We analyzed eight common steps in using an inhaler, including (i) opening; (ii) loading medication; (iii) positioning the device correctly or shaking it well; (iv) attaching the spacer; (v) breathing out gently; (vi) inhaling properly with a good seal; (vii-a) holding breath for about five seconds or as long as comfortable or (vii-b) breathing in and out normally for three or four breaths before removing the spacer from the mouth; and (viii) repeating the process as needed. All the critical steps in using an inhaler will be organized into the appropriate steps from 1 to 8. Information about these steps is shown in Supplementary Data.

### 2.6. Statistical Analysis

The statistical analysis was performed using STATA version 15.1 (StataCorp, College Station, TX, USA). Continuous variables were summarized as mean and standard deviation (SD) or median and interquartile range (IQR) for non-normally distributed data. Categorical variables were summarized as frequency counts and percentages. Fisher’s exact test, or the χ^2^ test, was used to compare the frequency of dichotomous variables. The Mann-Whitney-U test was used to compare continuous variables. The assessment of inter-rater reliability between two experts in the evaluation of the correct usage of inhalers can be achieved through Cohen’s Kappa coefficient, with 6 levels: 0 (no agreement), 0.10–0.20 (none to slight), 0.21–0.40 (fair), 0.41–0.60 (moderate), 0.61–0.80 (substantial), and 0.81–1.00 (almost perfect) [32]. In cases where two experts have different results regarding error identification, they will discuss together and reach a final decision. Variables have been incorporated into the multivariate analysis when the *p*-value in the univariate analysis is less than 0.2 [33,34]. This ensures that all relevant and potentially predictive variables are examined. The identical criterion was employed to identify which variables should be integrated into the derived model for this study. A *p*-value less than 0.05 was considered statistically significant.

### 2.7. Medical Ethics

The Ethical Review Committee at the University of Medicine and Pharmacy at Ho Chi Minh City provided approval for the present study (No. 567/HĐĐĐ-ĐHYD, 17 September 2020). All patients participating in the study signed informed consent.

## 3. Results

### 3.1. Patient Characteristics

The study involved a total of 91 patients with COPD over the age of 60. The mean age (±SD) was 77.3 ± 8.7. Males accounted for the majority of 85/91 cases (93.4%). The most common comorbidity was coronary artery disease, with a prevalence of 38.5%. Additional information regarding the baseline characteristics of the study population can be found in Table 1.

### 3.2. Characteristics Related to COPD

Overall, 91 patients had a median time [IQR] of COPD diagnosis until hospital admission of 4 [2,3,4,5,6] years. Most of these patients (86.8%) had “more breathlessness”, as indicated by a mMRC score ≥2. 41.8% of patients had a CAT score of ≥10. Over half of the patients had no exacerbations in the last 12 months. The average FEV_1_ was 60.4 ± 17.2%, with 63.7% of patients classified as GOLD 2. Overall, 78% of patients reported using more than one inhaler at home. However, only 41.8% of patients were directly instructed by healthcare staff on how to use inhalation devices correctly through technical demonstrations. More details about characteristics related to COPD are shown in Table 2. Moreover, regardless of the duration of inhaler usage, the rate of correct inhaler usage is not influenced by the duration of inhaler usage across all types of inhalers (*p* > 0.05) (Tables S1 and S2 in Supplementary Data).

### 3.3. The Rate of Correct Usage of Inhalers through Critical Steps

During the assessment of 133 records, two respiratory experts exhibited a high level of similarity in evaluating the number of errors, with only 15 cases showing any variation. This level of agreement was demonstrated by a Cohen’s Kappa coefficient of 0.88 (*p* < 0.001).

The most-used inhalers were pMDI and Respimat, accounting for 42/133 (31.6%) and 38/133 (28.6%), respectively. The correct usage rate of inhalers varied from 10.0% to 62.5%, with the exception of Accuhaler, as only one patient used it. Notably, out of all the patients, 17 demonstrated correct usage of all available inhalation devices, accounting for 18.7% of the total. The step of “holding breath for about five seconds or as long as comfortable” was performed incorrectly for pMDI, Respimat, Breezhaler, and Turbuhaler, with the rate of 81.0%, 50.0%, 66.7%, and 80.0%, respectively. The step “breathing out gently” also showed incorrect performance rates ranging from 63.2% to 80% for pMDI, Respimat, Breezhaler, and Turbuhaler. Details on the frequency of correct inhaler use for each type of inhaler and the percentage of critical steps performed incorrectly are shown in Table 3.

### 3.4. Univariate and Multivariable Analysis for Predicting Correct Inhaler Use

Univariate logistic regression was used to measure associations between age, sex, BMI, smoking, education level, comorbidity, duration of COPD, mMRC, CAT, the number of hospitalized exacerbations during the last 12 months, GOLD grades of airflow limitation, COPD group, length of hospital stay, the number of inhalers currently in use, and being instructed on how to use the inhaler between the two groups in terms of correctly using the inhaler (Table 4). Those variables with a *p*-value of <0.2 were included in a multivariable logistic regression model with a threshold *p*-value of 0.05. Multivariable logistic regression analysis showed that lower mMRC scores (AOR = 5.3, CI 1.1–25.5, *p* = 0.037) and receiving inhaler instruction within the past three months (AOR = 5.2, CI 1.3–20.1, *p* = 0.017) were associated with increased odds of using the inhaler correctly (Table 5).

## 4. Discussion

Our study revealed that the rate of correct usage of inhalers among patients with COPD over 60 is 18.7%. Two critical steps, “holding breath for about five seconds or as long as comfortable” and “breathing out gently”, are the most commonly misperformed steps when using pMDI, Respimat, Breezhaler, or Turbuhaler. A spacer can improve the accuracy of using pMDI or Respimat in older adults. Additionally, factors such as mMRC and being instructed on how to use the inhaler for the past three months increase the probability of correctly using inhalers by more than five times.

The rate of correct inhaler use in our study (18.7%) is relatively lower than Chau et al. [11] (24.3%), Chaicharn et al. [35] (25.2%), Melzer et al. [15] (34.5%), and Johanna et al. [36] (34.0%) (Table 6). Around 28% to 35% of individuals correctly use inhalers, and this rate has remained unchanged since 1975 in developed countries [10]. The higher rate of correct inhaler use in Johanna’s study may be due to only 4.5% of patients with COPD using pMDI in the study population [36]. In our study, pMDI was the most common inhaler with a very low accurate use rate (only 11.9% of cases). Melzer et al. [15] reported the correct usage rate of inhalers was 34.5%. However, the authors defined incorrect inhaler usage as making mistakes in more than 20% of the total inhaler-using steps (e.g., 2/11 steps for pMDI, 2/10 steps for Accuhaler, and 3/14 steps for Respimat). Our study uses the definition of “critical step”; if patients perform even one of the critical steps incorrectly, they will be considered misusing the inhaler. Because not all steps are significant and affect the patient outcome, it is essential to differentiate between “performing one step incorrectly” and “performing one critical step incorrectly” [31]. However, there is currently no consensus on the definition of “critical step”. A recent systematic review showed that there are over 30 different definitions of “critical step” for each type of inhaler. The critical step can be defined as a step that, when not performed or performed incorrectly, can affect the distribution of the drug to the airway [31]. Performing critical steps incorrectly may be associated with adverse outcomes such as increased symptom severity and frequency of exacerbations [37]. Our study is the first to apply the method of recording the process of patient inhaler use and then independently assessing the recording by two respiratory experts. Therefore, the reviewer can perform repeated assessments, calculating an accurate time (e.g., five seconds), and providing the most accurate conclusion after a consensus is reached between the two experts. We believe that the rate of correct inhaler use in clinical practice will be lower if we use a stricter definition such as “critical step” and have it evaluated by multiple respiratory experts.

Inhalers are the primary devices used to deliver medication in treating COPD, but their effectiveness in improving clinical outcomes may be diminished if patients do not use inhalers correctly [31]. Our study found that patients often perform two critical steps incorrectly when using inhalers without a spacer: “holding breath for about five seconds or as long as comfortable” and “breathing out gently”. This finding could direct the focus of healthcare providers to their essential instructions to increase the frequency of correct inhaler usage. Patients must hold their breath for five seconds since, during this step, medication particles will settle in the airways due to gravity, accounting for approximately 9% of the inhaled dose [39,40].

pMDI is widely used as a cheap device that can deliver different types of drugs for patients with COPD [41]. A study conducted in the United Kingdom showed that 15.8% of people over 70 were using an inhaler at home, with 42.8% using pMDI [42]. Our study revealed an approximate 75% rate of incorrect performance in the “breathing out gently” step, which is similar to Chau et al.’s study [11] (72.8%) but higher than Chaicharn et al.’s study [35] (54.5%). Although Chaicharn’s study was conducted on outpatients with COPD, our study and Chau’s were performed on hospitalized patients with COPD, which could impact the patient’s ability to complete this step accurately. Hospitalized patients often have more severe symptoms than outpatients, which may explain why inpatients cannot exhale completely to perform the “breathing out gently” step correctly. Moreover, our study uncovered a significant proportion of patients with COPD using pMDI who made mistakes in the step of “holding breath for about five seconds or as long as comfortable” with 81%, significantly higher than the studies by Chau and Chaicharn (34.9% and 27.3%, respectively). Although this assessment step is subjective and dependent on the reviewer, our study recorded the patients’ inhaler technique using video recordings, which facilitated the counting of seconds in this step. Dyspnea may hinder patients from completing a 5-second breath hold, resulting in a significant error in our study and increasing the likelihood of using the incorrect inhaler [43]. Therefore, our study shows that even with a very familiar device such as pMDI, the rate of patients misusing this inhaler is very high, especially among the elderly.

For Respimat, “breathing out gently” was the most frequent step performed incorrectly in our study. The studies conducted by Cruz [38] and Chau [11] also reached similar conclusions. For DPIs, regardless of the type of device, “breathing out gently” is still the most commonly performed step incorrectly in our study and those by Cruz [38], Chaicharn [35], and Chau [11]. Rootmensen’s study [44] found that the step “holding breath for five seconds or until comfortable” had high rates of incorrect performance for Breezhaler and Turbuhaler (51% and 80%, respectively), consistent with our study’s findings of 66.7% and 80% for these inhalers.

The technique of using an inhaler plays a crucial role in delivering the medication to the airway and depends on various factors such as experience, education level, the physical ability of the patient, and the proper instruction by healthcare staff [15]. Our study revealed that mMRC, and being instructed on how to use the inhaler for the past three months were independent predictors of correct inhaler use with the odds ratio (OR) from 5.2 to 5.3. Additionally, although not statistically significant, receiving instructions from healthcare staff on how to use an inhaler, accompanied by a demonstration, is associated with an increased likelihood of using the inhaler correctly (OR = 3.7, 95% CI 1.00–13.7, *p* = 0.051,). Table 7 demonstrates factors associated with correct inhaler use in several studies [11,35,36,44].

Johanna and Chaicharn’s study showed that patients with mild symptoms (as assessed by mMRC) tended to have a higher rate of correct inhaler use than those with more severe symptoms, although it was not statistically significant. Our study had similar findings. However, Chau reached a different conclusion, patients with lower mMRC scores were more likely to use the inhaler incorrectly, with an OR of 0.28 (95% CI 0.1–0.8; *p* < 0.05). Patients with COPD exacerbations are prone to cognitive decline, which may cause them to report their symptoms inaccurately [45]. Additionally, those with frequent exacerbations or hospitalizations in the past year tend to increase their perception of dyspnea more severely than those with less frequent exacerbations [43]. Therefore, whether the mMRC affects the rate of correct inhaler use in patients with COPD must be confirmed in future studies.

Our study found that patients who were instructed on how to use their inhalers within the past three months had 5.2 times higher odds of using their inhalers correctly compared to those who were not instructed or were instructed for more than three months. Chaicharn et al. [35] found no correlation between being instructed on inhaler use within the previous two years and correct inhaler usage. However, this may be due to patients forgetting or not maintaining the correct technique over a longer period. Studies have shown that after serial instructions for three months (once a month), the interval between each instruction session can be extended to three months without altering the patient’s outcome [46]. Our study supports this finding and can be applied in clinical practice, particularly in cases where not all patients can have monthly follow-up re-examinations. Screening patients who have been instructed for over three months can help physicians focus on instructing proper inhaler use, reducing the burden on healthcare personnel while ensuring appropriate treatment.

Furthermore, the quality of the instructions provided is also important. Our study has demonstrated that when patients receive instruction on inhaler usage with demonstrations from healthcare staff, their likelihood of using inhalers correctly increases significantly, with an odds ratio of 3.7. Therefore, in addition to adjusting techniques during instruction, demonstrating the correct usage of each device helps healthcare providers understand the challenges of operating inhalers and serves as a model for patients to observe and follow. Rootmensen et al. had a similar conclusion [44]. While the results are borderline, being instructed on how to use an inhaler with a demonstration by healthcare staff could be a key factor in prompting further studies to evaluate the specific responsibilities involved in instructing patients.

Our research has some limitations. Firstly, we excluded older patients with neurological and muscular disorders due to the study design, so we believe the actual correct rate of using inhaler would be even lower. Secondly, the mMRC score only assesses the degree of breathlessness and does not comprehensively reflect the overall impact of COPD. The GOLD guidelines recommend using multidimensional questionnaires, so studies on the relationship between multidimensional questionnaires and the adherence rate to inhaler use should be conducted. Thirdly, due to the limitation of capturing a single instance of inhaler use in the study, we were unable to differentiate between patients who consistently made errors and those who made occasional errors. Nevertheless, we mitigated this limitation by excluding patients with severe and unstable medical conditions, such as unimproved respiratory failure, acute myocardial infarction, acute stroke, or delirium. By implementing these exclusion criteria, we aimed to minimize the confounding effects of these conditions on the study outcomes. Lastly, because only one patient used Accuhaler in our study, we could not draw conclusions about this type of inhalation device.

## 5. Conclusions

Our study discovered that only approximately one-fifth of elderly patients with COPD use inhalers correctly. The two steps, “holding breath for about five seconds or as long as comfortable” and “breathing out gently”, are the most commonly performed incorrectly with different devices. Additionally, mMRC, and being instructed on how to use the inhaler for the past three months were independent predictors of correct inhaler use. These results can help clinical practitioners better manage elderly patients with COPD and improve the rate of correct inhaler use.

## Figures and Tables

**Table 1:** Baseline of the study population (N = 91).

Variables	N (%)	Mean ± SD
Age (years)		77.3 ± 8.7
60–69	19 (20.9)	
70–79	39 (42.9)	
≥80	33 (36.2)	
Sex		
Male	85 (93.4)	
Female	6 (6.6)	
BMI (kg/m^2^)		21.4 ± 3.8
Underweight (<18.5 kg/m^2^)	21 (23.1)	
Normal (18.5–22.9 kg/m^2^)	36 (39.6)	
Overweight (23–24.9 kg/m^2^)	22 (24.2)	
Obese (≥25 kg/m^2^)	12 13.2)	
Smoking	89 (97.8)	
Pack. years (among smokers)		39.9 ± 19.0
Education level		
Low-level	42 (46.1)	
Middle-level	38 (41.8)	
High-level	11 (12.1)	
Comorbidity		
Neuromuscular disease affects upper limbs *	8 (8.8)	
Asthma	9 (9.9)	
Bronchiectasis	10 (11.0)	
Chronic coronary syndromes	35 (38.5)	
Chronic heart failure	8 (8.8)	
Other condition		
Facial deformity **	0 (0.0)	

* Including 1 patient with a stiff shoulder, 4 patients with hemiplegia, 2 patients with Parkinson’s disease, and 1 patient with a one-finger hand. SD: standard deviation; BMI: body mass index. ** Including trauma, facial surgery, and congenital underdeveloped jaw.

**Table 2:** Characteristics related to chronic obstructive pulmonary disease (N = 91).

Variables	N (%)	Mean ± SD Median [IQR]
Duration (years)		4 [2–6]
≤1	11 (12.1)	
>1–5	50 (54.9)	
>5–10	26 (28.6)	
>10	4 (4.4)	
mMRC		
1	12 (13.2)	
2	47 (51.6)	
3	27 (29.7)	
4	5 (5.5)	
CAT		8 [6–12]
<10	53 (58.2)	
≥10	38 (41.8)	
The number of hospitalized exacerbations during the last 12 months		
0	50 (54.9)	
1	21 (23.1)	
2	9 (9.9)	
≥3	11 (12.1)	
FEV_1_ (%)		60.4 ± 17.2
GOLD grades of airflow limitation		
GOLD 1	9 (9.9)	
GOLD 2	58 (63.7)	
GOLD 3	19 (20.9)	
GOLD 4	5 (5.5)	
COPD groups		
A	4 (4.4)	
B	41 (45.1)	
E	46 (50.5)	
Length of hospital stay ≤ 7 days	45 (49.5)	
The number of inhalers currently in use *		
1	20 (22.0)	
2	55 (60.4)	
≥3	16 (17.6)	
Be instructed on how to use inhaler		
For the past 3 months	30 (33.0)	
More than 3 times in the past year	6 (6.6)	
With demonstration by healthcare staff	38 (41.8)	

* The inhalers can be of the same type. SD: standard deviation; COPD: chronic obstructive pulmonary disease; mMRC: modified medical research council; CAT: COPD assessment test; FEV_1_: forced expiratory volume in the first second; GOLD: global initiative for chronic obstructive lung disease; IQR: interquartile range.

**Table 3:** The rate of correct usage of inhalers through critical steps (N = 133).

Type of Inhaler Device *	I	II	III	IV	V	VI	VII
Number of inhaler usage (n)	42	8	38	13	21	10	1
Correct inhaler usage (n, %)	5 (11.9)	5 (62.5)	11 (28.9)	4 (30.8)	5 (23.8)	1 (10.0)	1 (100)
The frequency of performing the steps incorrectly ** (n, %)							
Opening	0 (0)	0 (0)	6 (15.8)	7 (53.8)	0 (0)	0 (0)	0 (0)
Loading medication	--	--	--	--	1 (4.8)	--	0 (0)
Positioning the device correctly/shake well	7(16.7)	3 (37.5)	--	--	--	3 (30.0)	--
Attaching spacer	--	0 (0)	--	0 (0)	--	--	--
Breathing out gently	32 (76.2)	--	24 (63.2)	--	14 (66.7)	8 (80.0)	0 (0)
Inhaling properly with a good seal	29 (69.0)	1(12.5)	14 (36.8)	1 (7.7)	7 (33.3)	3 (30.0)	0 (0)
Holding breath for about 5 s or as long as comfortable	34 (81.0)	0 (0)	19 (50.0)	0 (0)	14 (66.7)	8 (80.0)	0 (0)
Breathing in and out normally for 3 or 4 breaths before removing spacer from the mouth	--	0 (0)	--	0 (0)	--	--	--
Repeating as needed	20 (47.6)	--	0 (0)	1 (7.7)	1 (4.8)	4 (40.0)	0 (0)

* I: pMDI; II: pMDI spacer plus; III: Respimat; IV: Respimat spacer plus; V: Breezhaler; VI: Turbuhaler; VII: Accuhaler. ** Critical steps can be found in supplementary data.

**Table 4:** Clinical and laboratory characteristics between the correct inhaler use group and the incorrect inhaler use group (N = 91).

Variables	All N (%) Mean ± SD Median [IQR]	Correct Inhaler Use N (%) Mean ± SD Median [IQR]	Incorrect Inhaler Use N (%) Mean ± SD Median [IQR]	*p*
Sex (male)	85 (93.4)	17 (100)	68 (91.9)	0.22 *
Age (years)	77.3 ± 8.7	79.1 ± 10.5	76.9 ± 8.3	0.51 ^+^
BMI (kg/m^2^)	21.4 ± 3.8	21.7 ± 3.7	21.3 ± 3.8	0.55 ^+^
Smoking	89 (97.8)	17 (100)	72 (97.3)	1.00 *
Education level				
Low-level	42 (46.2)	7 (41.2)	35 (47.3)	0.93 *
Middle-level	38 (41.8)	8 (47.1)	30 (40.5)	
High-level	11 (12.1)	2 (11.8)	9 (12.2)	
Comorbidity				
Neuromuscular disease affects upper limbs	8 (8.8)	1 (5.9)	7 (9.5)	1.00 *
Asthma	9 (9.9)	3 (33.3)	6 (66.7)	0.36 *
Bronchiectasis	10 (11.2)	3 (17.6)	7 (9.5)	0.40 *
Chronic coronary syndromes	35 (38.5)	9 (52.9)	26 (35.1)	0.27 *
Chronic heart failure	8 (8.8)	2 (11.8)	6 (8.1)	0.64 *
Duration (years)	4 [3–6]	5 [2.5–8]	4 [2–6]	0.38 ^++^
mMRC				
0–1	12 (13.2)	5 (29.4)	7 (9.5)	0.04 *
2–4	79 (86.8)	12 (70.6)	67 (90.5)	
CAT				
<10	53 (58.2)	10 (58.8)	43 (58.1)	1.00 ^+^
≥10	38 (41.8)	7 (41.2)	31 (41.9)	
The number of hospitalized exacerbations during the last 12 months				
0	50 (54.9)	8 (47.1)	42 (56.8)	0.53 *
1	21 (23.1)	6 (35.3)	15 (20.3)	
2	9 (9.9)	2 (11.8)	7 (9.5)	
≥3	11 (12.1)	1 (5.9)	10 (13.5)	
GOLD grades of airflow limitation				
1–2	67 (73.6)	15 (88.2)	52 (70.3)	0.13 *
3–4	24 (27.4)	2 (11.8)	22 (29.7)	
COPD groups				
A	4 (4.4)	2 (11.8)	2 (2.7)	0.12 *
B	41 (45.1)	5 (29.4)	36 (48.7)	
E	46 (50.5)	10 (58.8)	36 (48.6)	
Length of hospital stay ≤ 7 days	45 (49.5)	5 (29.4)	40 (54.1)	0.11 *
The number of inhalers currently in use				
1	20 (22.0)	4 (23.5)	16 (21.6)	1.00 *
2	55 (60.4)	10 (58.8)	45 (60.8)	
3	16 (17.6)	3 (17.7)	13 (17.6)	
Be instructed on how to use inhaler				
For the past 3 months	30 (33.0)	12 (70.6)	18 (24.3)	<0.01 *
More than 3 times in the past year	6 (6.6)	3 (17.7)	3 (4.0)	0.08 *
With demonstration by healthcare staff	38 (41.8)	11 (64.7)	47 (63.5)	0.05 *

* Fisher’s exact test. ^+^ *t*-student test. ^++^ Mann-Whitney-U test. SD: standard deviation; COPD: chronic obstructive pulmonary disease; mMRC: modified medical research council; CAT: COPD assessment test; GOLD: global initiative for chronic obstructive lung disease; BMI: body mass index; IQR: interquartile range.

**Table 5:** Univariate and multivariable analysis for predicting correct inhaler use.

Variable	Model 1 *		Model 2 **		
	OR	*p*	AOR	95% CI	*p*
mMRC	4.0	0.04	5.3	1.1–25.5	**0.037 ^†^**
GOLD grades of airflow limitation	3.2	0.13	2.4	0.4–15.1	0.336
Length of hospital stay ≤7 days	2.8	0.11	2.3	0.6–9.2	0.251
Be instructed on how to use inhaler					
For the past 3 months	7.5	0.001	5.2	1.3–20.1	**0.017 ^†^**
More than 3 times in the past year	5.1	0.06	2.9	0.4–22.2	0.305
With demonstration by healthcare staff	3.2	0.04	3.7	1.00–13.7	0.051

* Model 1: univariate logistic regression analysis. ** Model 2: multivariable logistic regression analysis. † *p*-value < 0.05. OR: odds ratio; AOR: adjusted odds ratio; CI: confidence interval; mMRC: modified medical research council; GOLD: Global Initiative for Chronic Obstructive Lung Disease.

**Table 6:** The rate of correct inhaler usage in several studies.

Study	Rate of Correct Inhaler Use (%)	pMDI (Spacer Plus) (%)	SMI (Spacer Plus) (%)	DPI (%)	Br (%)	Tu (%)	Ac (%)
Chaicharn [35]	25.2	22.7 (30.3)					26.1
Melzer [15]	34.5	34.3					50.5
Cruz [38]			36.3		40.4		
Chau [11]	24.3	22.7	31.8	30.4			
Johanna [36]	34.0		61.0		64.0	45.0	10.0
Our study	18.7	11.9 (62.5)	28.9 (30.8)	21.9	23.8	10.0	100

pMDI: pressurized metered dose inhaler; SMI: soft mist inhaler; DPI: dry powder inhaler, Br: Breezhaler; Tu: Turbuhaler; Ac: Accuhaler.

**Table 7:** Factors associated with correct inhaler use in several studies.

Factors	Chau [11]	Johanna [36]	Chaicharn [35]	Rootmensen [44]	Our Study
mMRC (mild symptoms)					
OR	0.28	1.14	1.6	--	5.3 *
95% CI	0.1–0.8	0.7–1.8	0.7–4.2	--	1.1–25.5
GOLD grades of airflow limitation					
OR	--	--	1.4	1.2	2.4
95% CI	--	--	0.5–3.3	0.6–2.4	0.4–15.1
Length of hospital stay					
OR	2	--	--	--	2.3
95% CI	1.1–3.6	--	--	--	0.6–9.2
Be instructed on how to use inhaler for the past 3 months					
OR	--	--	1.1 **	--	5.2 *
95% CI	--	--	0.5–2.8	--	1.3–20.1
Be instructed on how to use inhaler more than 3 times in the past year					
OR	--	--	--	--	2.9
95% CI	--	--	--	--	0.4–22.2
Be instructed on how to use inhaler with demonstration by healthcare staff					
OR	--	--	--	2.2	3.7
95% CI	--	--	--	1.02–4.8	1.00–13.7

* *p*-value < 0.05. ** Be instructed on how to use inhalers for the last 2 years. OR: odds ratio; CI: confidence interval; mMRC: modified medical research council; GOLD: Global Initiative for Chronic Obstructive Lung Disease.

## Data Availability

The datasets generated during and/or analyzed during the current study are available from the corresponding author on reasonable request.

## Supplementary Materials

The following supporting information can be downloaded at: https://www.mdpi.com/article/10.3390/jcm12134420/s1, Table S1: pMDI and pMDI plus spacer checklists, Table S2: Respimat and Respimat plus spacer checklists, Table S3: Breezhaler, Turbuhaler and Accuhaler checklists, Table S4: The median duration of inhaler usage for each type of inhaler, Table S5: The correlation between the duration of inhaler usage and the rate of correct inhaler usage within each type of inhaler.
