# Supplementary material for: Prevalence and Factors Affecting Appropriate Inhaler Use in Elderly Patients with Chronic Obstructive Pulmonary Disease: A Prospective Study
Source: J Clin Med. 2023 Jun 30;12(13):4420. doi: 10.3390/jcm12134420 (PMC10342446; doi:10.3390/jcm12134420)
Supplement: Supplementary file 1 [file jcm-12-04420-s001.zip › jcm-2435967-supplementary.pdf]

Table S1: pMDI and pMDI plus spacer checklists

8 steps used for analysis		pMDI*	pMDI plus spacer*
1	Open	Remove inhaler cap	Remove inhaler cap
2	Load medication		
3	Position the device correctly/shake well	Hold inhaler upright and shake well	Hold inhaler upright and shake well before inserting into spacer
4	Attach spacer		Put mouthpiece between teeth without biting and close lips to form a good seal
5	Breathe out gently	Breathe out gently, away from the inhaler	
6	Inhale properly with a good seal	Breathe in slowly through the mouth and, at the same time, press down firmly on canister	Hold spacer level and press down firmly on inhaler canister once
7a	Hold breath for about 5 seconds or as long as comfortable	Keep breathing in slowly and deeply and hold breath for about 5 seconds or as long as comfortable	Single breath: Breathe in slowly and deeply and hold breath for around 5 seconds or as long as comfortable. Take spacer out of mouth while holding breath
7b	Breathe in and out normally for 3 or 4 breaths before removing spacer		Tidal breath: Breathe in and out normally for 3 or 4 breaths before removing spacer from the mouth

	from the mouth		
8	Repeat as needed	If an extra dose is needed, repeat steps 2 to 8	

** All steps listed are critical steps. The inhaler technique checklists can be found online at the following website: https://www.nationalasthma.org.au/living-with-asthma/resources/health-professionals/charts/inhaler-technique-checklists (accessed on 20 May 2023).*

Table S2: Respimat and Respimat plus spacer checklists

8 steps used for analysis		Respimat*	Respimat plus spacer*
1	Open	Turn base in direction of arrows until it clicks (half a turn)<br>Open the cap until it snaps fully open	Turn base in direction of arrows until it clicks (half a turn)<br>Open the cap until it snaps fully open
2	Load medication		
3	Position the device correctly/shake well		
4	Attach spacer		Put mouthpiece between teeth without biting and close lips to form a good seal
5	Breathe out gently	Breathe out gently, away from inhaler	
6	Inhale properly with a good seal	Breathe in slowly and deeply through mouth and, at the same time, press down on the dose button	Hold spacer level and press down on the dose button once
7a	Hold breath for about 5 seconds or as long as comfortable	Hold breath for 5 seconds or as long as comfortable	Single breath: Breathe in slowly and deeply and hold breath for around 5 seconds or as long as comfortable. Take spacer out of mouth while holding breath

7b	Breathe in and out normally for 3 or 4 breaths before removing spacer from the mouth		Tidal breath: Breath in and out normally for 3 or 4 breaths before removing spacer from the mouth
8	Repeat as needed	Repeat from step 1 to get the full dose (as two inhalations is the usual dose for medicines used with Respimat)	If an extra dose is needed, repeat steps 3 to 9

** All steps listed are critical steps. The inhaler technique checklists can be found online at the following website: https://www.nationalasthma.org.au/living-with-asthma/resources/health-professionals/charts/inhaler-technique-checklists (accessed on 20 May 2023).*

Table S3: Breezhaler, Turbuhaler and Accuhaler checklists

8 steps used for analysis		Breezhaler*	Turbuhaler*	Accuhaler*
1	Open	Remove cap	Unscrew and remove cover	Open cover using thumb grip
2	Load medication	Remove capsule from blister and place in chamber<br>Close mouthpiece until it clicks<br>Press side buttons in once and release (do not shake)		Hold horizontally, load dose by sliding lever until it clicks
3	Position the device correctly/shake well		Keep inhaler upright while twisting grip<br>Twist around and then back until click is heard	
4	Attach spacer			
5	Breathe out gently	Breathe out gently, away from inhaler	Breathe out gently, away from the inhaler	Breathe out gently, away from the inhaler
6	Inhale properly with a good seal	Breathe in quickly and	Breathe in strongly and	Breathe in steadily and

		steadily, so capsule vibrates	deeply	deeply
7 a	Hold breath for about 5 seconds or as long as comfortable	Hold breath for about 5 seconds, or as long as comfortable	Hold breath for about 5 seconds or as long as comfortable	Hold breath for about 5 seconds or as long as comfortable<br>While holding breath, remove inhaler from mouth
7 b	Breathe in and out normally for 3 or 4 breaths before removing spacer from the mouth			
8	8. Repeat as needed	If more than one dose is needed, repeat steps 3 to 12	If an extra dose is needed, repeat steps 2 to 9	If an extra dose is needed repeat steps 2 to 8

** All steps listed are critical steps. The inhaler technique checklists can be found online at the following website: https://www.nationalasthma.org.au/living-with-asthma/resources/health-professionals/charts/inhaler-technique-checklists (accessed on 20 May 2023)*

Table S4: The median duration of inhaler usage for each type of inhaler

Inhaler	Observation (n)	Median (years)	IQR (years)
pMDI	42	3	2-5
pMDI plus spacer	8	1.5	0.5-2
Respimat	38	2	0.8-3
Respimat plus spacer	13	1	0.5-2
Breezhaler	21	1	0.3-2
Turbuhaler	10	2	0.1-3
Accuhaler	1	5	

In the case of a patient using multiple inhalers of the same type, we prioritize the inhaler with the longest duration of use.

Abbreviation: pMDI, pressured metered dose inhaler; IQR, interquartile range.

Table S5: The correlation between the duration of inhaler usage and the rate of correct inhaler usage within each type of inhaler

Inhaler	Observation (n)	Time (years [IQR])		*p**
		Correct	Incorrect	
pMDI	42	3.5 (1.5-6.5)	3.0 (2.0-5.0)	0.98
pMDI plus spacer	8	1.0 (0.5-2.0)	2.0 (0.5-2.0)	0.46
Respimat	38	2.0 (1.0-3.0)	2.0 (0.5-3.0)	0.57
Respimat plus spacer	13	0.6 (0.1-2.0)	1.5 (0.5-2.0)	0.94
Breezhaler	21	1.1 (0.2-2.0)	1.0 (0.3-2.0)	0.25
Turbuhaler	10	0.1 (0.1-2.0)	2.0 (0.5-4.0)	0.94

*Mann-Whitney-U test.

Abbreviation: pMDI, pressured metered dose inhaler; IQR, interquartile range.
